# Delayed Comparison and Apriori Algorithm (DCAA): A Tool for Discovering Protein–Protein Interactions From Time-Series Phosphoproteomic Data

**DOI:** 10.3389/fmolb.2020.606570

**Published:** 2020-12-10

**Authors:** Lianhong Ding, Shaoshuai Xie, Shucui Zhang, Hangyu Shen, Huaqiang Zhong, Daoyuan Li, Peng Shi, Lianli Chi, Qunye Zhang

**Affiliations:** ^1^School of Information, Beijing Wuzi University, Beijing, China; ^2^National Glycoengineering Research Center, Shandong University, Qingdao, China; ^3^The Key Laboratory of Cardiovascular Remodeling and Function Research, Chinese Ministry of Education, Chinese National Health Commission and Chinese Academy of Medical Sciences, Qilu Hospital of Shandong University, Jinan, China; ^4^National Center for Materials Service Safety, University of Science and Technology Beijing, Beijing, China

**Keywords:** protein–protein interactions, phosphoproteomics, delayed comparison, Apriori, DCAA

## Abstract

Analysis of high-throughput omics data is one of the most important approaches for obtaining information regarding interactions between proteins/genes. Time-series omics data are a series of omics data points indexed in time order and normally contain more abundant information about the interactions between biological macromolecules than static omics data. In addition, phosphorylation is a key posttranslational modification (PTM) that is indicative of possible protein function changes in cellular processes. Analysis of time-series phosphoproteomic data should provide more meaningful information about protein interactions. However, although many algorithms, databases, and websites have been developed to analyze omics data, the tools dedicated to discovering molecular interactions from time-series omics data, especially from time-series phosphoproteomic data, are still scarce. Moreover, most reported tools ignore the lag between functional alterations and the corresponding changes in protein synthesis/PTM and are highly dependent on previous knowledge, resulting in high false-positive rates and difficulties in finding newly discovered protein–protein interactions (PPIs). Therefore, in the present study, we developed a new method to discover protein–protein interactions with the delayed comparison and Apriori algorithm (DCAA) to address the aforementioned problems. DCAA is based on the idea that there is a lag between functional alterations and the corresponding changes in protein synthesis/PTM. The Apriori algorithm was used to mine association rules from the relationships between items in a dataset and find PPIs based on time-series phosphoproteomic data. The advantage of DCAA is that it does not rely on previous knowledge and the PPI database. The analysis of actual time-series phosphoproteomic data showed that more than 68% of the protein interactions/regulatory relationships predicted by DCAA were accurate. As an analytical tool for PPIs that does not rely on a priori knowledge, DCAA should be useful to predict PPIs from time-series omics data, and this approach is not limited to phosphoproteomic data.

## Introduction

Protein–protein interactions (PPIs) are the basis and prerequisite for protein functions. Proteins *in vivo* are part of complex regulatory networks involving sophisticated interactions to coordinately regulate various biological processes and functions under different spatiotemporal conditions. The PPIs in living organisms are more complex than one might imagine. Therefore, PPI is one of the most critical issues in biomedical research (Braun and Gingras, 2012). Many experimental techniques and equipment for studying PPIs have been developed, such as immunoprecipitation, biolayer interferometry, and surface plasmon resonance (Douzi, 2017; Lin and Lai, 2017; Wu et al., 2017). Although these techniques are reliable and widely used, they are time- and cost-consuming and low-throughput. To reveal the mechanisms underlying physiological and pathological processes, high-throughput methods for studying PPIs are urgently needed. To date, some high-throughput experimental methods for detecting PPIs have been reported, such as yeast two-hybrid, tandem affinity purification, phage display, and protein chip methods (Gavin et al., 2002; Rao et al., 2014; Sundell and Ivarsson, 2014; Mehla et al., 2015; Huang et al., 2017; Viala and Bouveret, 2017; Woloschuk et al., 2020). However, these methods also have many drawbacks, including complexity, required time, and high cost. Therefore, computational methods could be useful supplements to high-throughput experimental methods (Lei et al., 2016; Sun et al., 2017; Zhang et al., 2017).

In the past two decades, high-throughput omics technologies, including genomes, transcriptomes and proteomes, have developed rapidly (Mortazavi et al., 2008; Consortium, 2012; Liu et al., 2018; Jiang et al., 2019). Likewise, many tools have been developed to analyze these omics data and obtain useful information about protein/gene interactions. For example, BindML+ can predict PPIs using an amino acid substitution model, and PIC (Protein Interaction Calculator) is a web tool to compute intra- and interprotein interactions (Tina et al., 2007; La et al., 2013). Time-series omics data are a series of omics data points indexed in time order and normally contain more abundant information about the interactions between biological macromolecules than static omics data. Therefore, many tools and websites have been proposed for discovering PPIs based on these data. To date, the algorithms used in the reported tools include learning vector quantization (LVQ), profile-kernel support vector machine, random forest classifier, semantic-based regularization (a machine learning framework), feature extraction, and deep learning (Planas-Iglesias et al., 2013; Yousef and Moghadam Charkari, 2013; Saccà et al., 2014; Hamp and Rost, 2015; Sun et al., 2017; Zeng et al., 2020). However, many of the reported tools require substantial amounts of supporting data in addition to omics data, such as protein structure, protein/gene sequence, gene functional similarity, and protein–protein interaction databases (Planas-Iglesias et al., 2013; Sun et al., 2017; Zeng et al., 2020).

Phosphorylation is the most common posttranslational modification (PTM) of proteins for functional regulation (Cohen, 2000; Ardito et al., 2017). Thus, phosphoproteomic data might suggest possible changes in protein function. Analysis of time-series phosphoproteomic data should provide more meaningful information about protein interactions. However, although many algorithms, databases, and websites have been developed to analyze omics data, the tools dedicated to discovering molecular interactions from time-series omics data, especially from time-series phosphoproteomic data, are still scarce. More importantly, protein interactions and PTMs are a series of events that undergo sequential and dynamic alterations. There are lags between functional alterations and the corresponding changes in protein synthesis/PTM. However, the most widely reported tools ignore these lags and are highly dependent on previous knowledge, resulting in high false-positive rates and difficulties in finding newly discovered PPIs. In addition, the false-positive rates of PPIs predicted by many tools from static omics data are very high.

In this study, considering the aforementioned lags, we propose a novel method for predicting PPIs combining delayed comparison and the Apriori algorithm (DCAA), which does not rely on previous knowledge. High-throughput dynamic phosphoproteomic data from human umbilical vein endothelial cells treated with oxidized low-density lipoprotein (ox-LDL) were used to verify this method. By not relying on previous knowledge and the PPI database, DCAA could discover PPIs from dynamic phosphoproteomic data with a relatively low false-positive rate. Moreover, DCAA should also be applied to other time-series omics data.

## Materials and Methods

### Cell Treatment and Protein Digestion by Trypsin

EA.hy926 cells were purchased from the American Tissue Culture Collection (Manassas, USA) and cultured in Dulbecco's modified essential medium containing 10% fetal bovine serum (FBS), 100 U/ml penicillin G, and 100 μg/ml streptomycin. After treatment with 50 μg/ml ox-LDL for 0, 0.5, 1, 1.5, 2, 4, 6, 8, 12, 18, 24, 36, 48, and 72 h, all cells were harvested and lysed in lysis buffer (8 M urea, 50 mM Tris–HCl, 10% isopropyl alcohol, 12.5% isobutyl alcohol containing complete protease inhibitor cocktail and PhosSTOP phosphatase inhibitor cocktail). The common control samples were produced by mixing equal amounts of all 14 samples. All samples (2 common controls and 14 time point samples, 400 μg/sample) were reduced with dithiothreitol for 1 h after alkylation by iodoacetamide for 1 h in the dark. After replacing the solvent with 50 mM triethylammonium bicarbonate using ultracentrifugal filtration units (MWCO 10 kDa), all samples were digested by trypsin at 37°C for 18 h with a 50:1 protein-to-protease ratio.

### Phosphopeptide Enrichment and iTRAQ Labeling

The tryptic digests of all 16 samples were labeled with 8-Plex iTRAQ (SCIEX, MA, USA) according to the manufacturer's instructions. Then, the 16 samples were equally divided into two sample pools. Each sample pool contained equal amounts of proteins from a common control (labeled with iTRAQ 113) and seven time point samples (from 0 to 6 h, respectively labeled with iTRAQ 114-iTRAQ 121; or from 8 to 72 h, respectively labeled with iTRAQ 114-iTRAQ 121). After desalting and drying, the peptides from two sample pools were dissolved in 5 ml of TiO_2_ loading buffer (1.25 M glycolic acid, 80% ACN, 1% TFA) and incubated with 16 mg of Titanosphere TiO_2_ (5 μm; GL Science, Tokyo, Japan) for 30 min. The TiO_2_ beads were washed sequentially with TiO_2_ loading buffer, 1% TFA in 80% aqueous ACN, and 0.1% TFA in 2% aqueous ACN. Then, the beads were eluted sequentially with 8% NH_4_OH and 50 mM phosphate buffer (pH 12.0). Finally, the eluates were combined and immediately neutralized with 10% FA.

### Peptide Fractionation Using Basic Reversed-Phase Liquid Chromatography

After neutralization, each sample pool was dissolved in 80 μl of buffer A (2% ACN, 15 mM NH_4_COOH, pH 10.0) and separated using basic reversed-phase liquid chromatography at 0.14 ml/min with a Kinetex EVO C18 column (2.6 μm particles, 100 Å, 15 cm × 2.1 mm; Phenomenex, Torrance, USA). For separation, a step gradient of 2% B (80% ACN, 15 mM NH_4_COOH, pH 10.0), 0–8 min; 2–28% B, 8–68 min; 28–40% B, 68–78 min; 40–100% B, 78–83 min; and 100% B, 83–93 min was used. The eluates were collected at 1-min intervals and then pooled into 16 fractions. After desalting and drying, the fractions were dissolved in 0.1% FA for LC-MS/MS analysis.

### Nano-LC-MS/MS Analysis

Each sample pool was loaded on a ReproSil-Pur C18 precolumn (3 cm × 100 μm, 5 μm, 120 Å; Dr Maisch, Germany) at 5 μl/min using an Easy nLC-1000 nano-LC system (Thermo Scientific, San Jose, CA, USA). For separation, mobile phase A was 0.1% FA in 2% ACN, and mobile phase B was 0.1% FA in 98% ACN. A step gradient of 2–8% B, 0–5 min; 8–22% B, 5–85 min; 22–30% B, 85–105 min; 30–90% B, 105–110 min; and 90% B, 110–120 min was used at 300 nl/min. Data-dependent MS/MS was performed using an Orbitrap Fusion mass spectrometer in positive ion mode with the following parameters: 2.2 kV spray voltage, 275°C capillary temperature, 55% S-lens level, 350–1,550 mass acquisition range, and 120,000 resolution for MS analysis. Each precursor ion scan was followed by a 4-s top speed data-dependent HCD MS/MS at 35% normalized collision energy. The resolution for MS/MS analysis was 30,000. The quadrupole isolation width was 2 *m*/*z*. The dynamic exclusion time was 60 s with a ±10 ppm exclusion mass width. The raw data were processed using Proteome Discoverer version 1.4.0.28 and the UniProt database. The PhosphoRS 3.0 algorithm was used to evaluate the localization probabilities of phosphorylation sites.

### Clustering Process of the DCAA Method

To reduce the data volume for better processing, the original data consisting of the relative expression levels of phosphopeptides were first clustered according to shape similarity, namely, based on the well-known fact that trends of greater data similarity indicate closer relationships between data. Three clustering methods, including full trend clustering, angle clustering, and Pearson clustering, were used in the present study. Full trend clustering classifies the original data according to the changing trend (increasing, decreasing, or unchanging) of every line segment, which is constructed with two close time points of the relative expression data of phosphopeptides. The phosphopeptides with the same changing trends are classified into one cluster. The angle clustering classifies the original data considering both the speed and trend of the changes in the relative expression of phosphopeptides. The change speeds are measured by the angle between two-line segments constructed as mentioned previously. The phosphopeptides with angles less than a given value are classified into one cluster. Pearson clustering classifies the original data based on the Pearson correlation coefficient of the relative expression of phosphopeptides, which indicates the degree of linear correlation. The Pearson correlation coefficient between vectors *X* and *Y* is defined as follows:

(1) $$\begin{array}{l} \rho_{X,Y}=\frac{\text{cov}(X,Y)}{\delta_{X}\delta_{Y}}=\frac{\sum_{\;}^{\;}XY-\frac{\sum_{\;}^{\;}X\sum_{\;}^{\;}Y}{N}}{\sqrt{\left(\sum_{\;}^{\;}X^{2}-\frac{{\left(\sum_{\;}^{\;}X\right)}^{2}}{N}\right)\left(\sum_{\;}^{\;}Y^{2}-\frac{{\left(\sum_{\;}^{\;}Y\right)}^{2}}{N}\right)}} \end{array}$$

where *N* is the dimension number of vectors *X* and *Y*.

### The Delayed Comparison Processes of the DCAA Method

Delayed comparison and shopping basket dataset construction were used to reflect the lags between upstream and downstream events of PPIs. The shopping basket dataset is named to reflect the concept of shopping in supermarkets in the data mining area. The data selection process is analogized as a good selection process in supermarkets. The shopping basket dataset is the set containing the selected data from all candidate data. There were 13 different time-points of phosphoprotein change data compared with the data at 0 h. Normally, the lags between upstream and downstream PPIs did not last very long. Therefore, we fixed nine time periods to build sliding time windows and control the delayed time periods within three time periods. The steps of delayed comparison are as follows:

a) Establish representatives of classes: the representative of a class was the arithmetic average of all the data in this class.
b) Convert data points into time periods: two adjacent data points were subtracted (*T*_1_ = *t*_2_-*t*_1_, *T*_2_ = *t*_3_-*t*_2_, …, *T*_12_ = *t*_13_-*t*_12_), and the values of time periods were obtained (*T*_1_, *T*_2_, …, *T*_12_).
c) Use delayed comparison: to fix time periods, a start time and delayed time periods were chosen. For example, 9.1.2 means there are 9 time periods, the start time is 1 and there are 2 delayed time periods. Sliding windows of six groups (9.1.1, 9.1.2, 9.1.3, 9.2.1, 9.2.2, and 9.3.1.) will cover all delayed scenarios.
d) Construct the shopping basket dataset: one cluster of original data was compared with another cluster of original data after sliding to achieve delayed comparison, thereby producing one shopping basket data item. Six shopping basket datasets obtained by delayed comparison were aggregated into one dataset to build the experimental dataset for the Apriori algorithm.

### The Processes of the Apriori Algorithm in the DCAA Method

The Apriori algorithm is a kind of machine learning algorithm, and its core purpose is to mine frequent sets of customer shopping records and excavate association rules (Agrawal et al., 1993). The nonempty subset of a frequent item set must be a frequent item set. The Apriori algorithm first generates one frequent item sets and then uses one frequent item sets to generate two frequent item sets. Next, three frequent item sets are generated from two frequent item sets. Finally, all frequent item sets are generated. Then, the association rules are found from these frequent item sets. The Apriori algorithm is outlined in Appendix 1. The confidence and minimum support of the Apriori algorithm are set up for obtaining interclass inference results. The support degree and confidence degree of an association rule between *X* and *Y* are, respectively, as follows:

(2) $$\begin{array}{l} \text{Support}\left(X,Y\right)=P\left(XY\right)=\frac{\text{number}\left(XY\right)}{\text{num}\left(\text{All Samples}\right)} \end{array}$$

(3) $$\begin{array}{l} \text{Confidence}\left(\text{X}⇐\text{Y}\right)=P\left(X|Y\right)=P\left(XY\right)/P\left(Y\right) \end{array}$$

**Figure 1:**
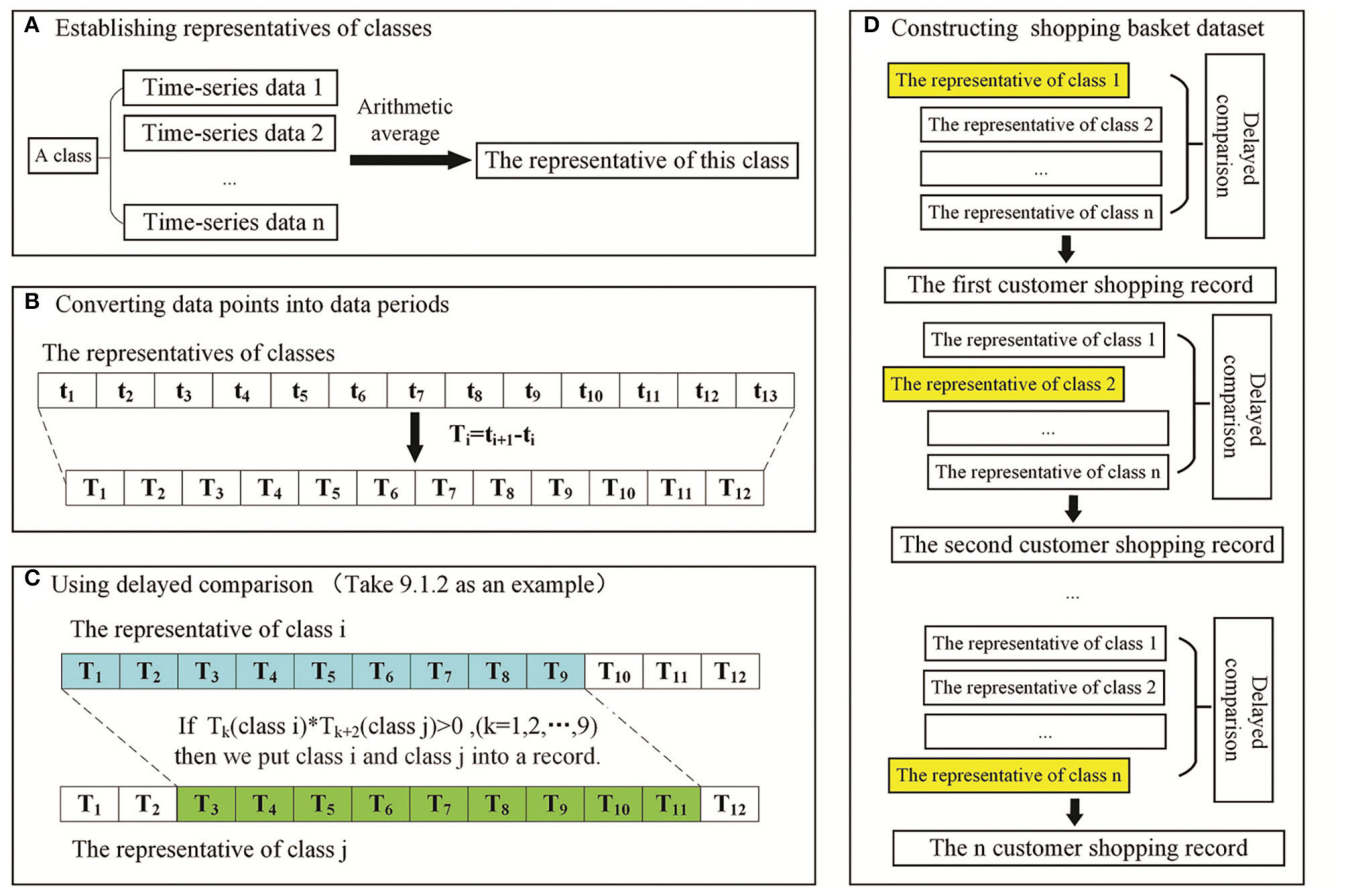
Schematic of delayed comparison to build the shopping basket dataset. **(A)** Representative establishment of a cluster. **(B)** Data points were subtracted to convert data points into time periods. **(C)** Delayed comparison for addressing the lags between functional alterations and their corresponding changes in protein synthesis/PTMs. **(D)** Construction of the shopping basket dataset for the Apriori algorithm.

An example of building a shopping basket dataset with a delayed comparison of 9.1.2 is shown in Figure 1. A customer shopping record was constructed as follows: *T*_*k*_ (class *i*)^*^T_*k*__+2_ (class *j*) > 0 or *T*_*k*_ (class *i*)=0 and *T*_*k*__+2_(class *j*)=0 (*k* = 1, 2, …, 9), class *i* and class *j* are put into one record. After applying the sliding window treatment described previously once, *n* (*n* ≥ 0) customer shopping records can be produced. Six shopping basket datasets are obtained using six kinds of sliding windows. They are combined into one dataset as the experimental dataset of the Apriori algorithm.

### Matching Process of the DCAA Method

**Figure 2:**
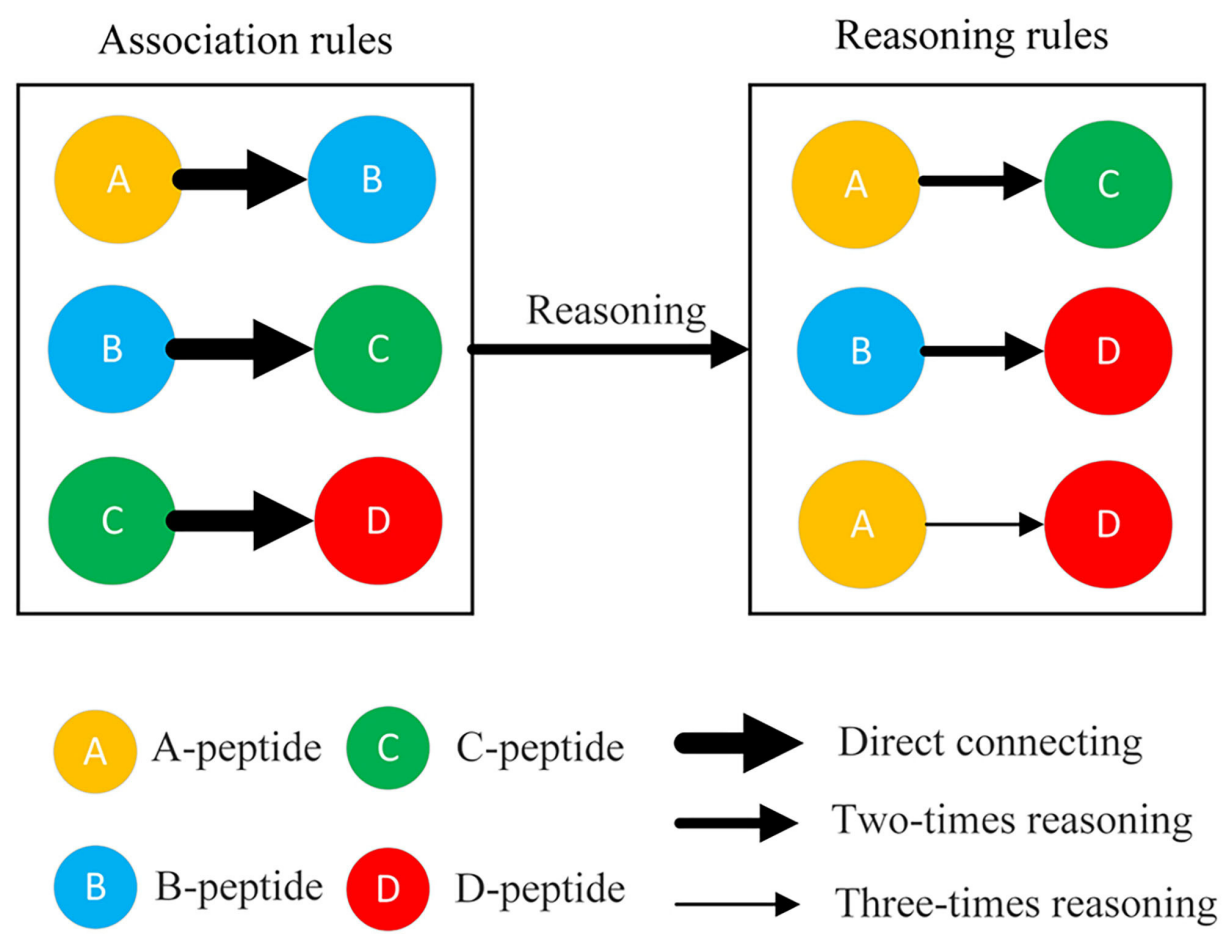
Three rules for matching association. The connections of two peptides were divided into three types: direct connection, twofold reasoning sessions, and threefold reasoning sessions. Direct connection (one-time reasoning): two phosphopeptides/proteins were directly linked. Twofold reasoning: two phosphopeptides/proteins were linked through a different phosphopeptide/protein. Threefold reasoning: two phosphopeptides/proteins were linked through two different phosphopeptides/proteins.

The association rules among phosphopeptides were discovered from the delayed comparison and Apriori algorithm results using different reasoning methods, including direct relationship (one-time reasoning), two-times reasoning, and three-times reasoning (Figure 2). One-time reasoning means that two phosphopeptides/proteins are directly linked. Two-times reasoning means that two phosphopeptides/proteins are linked through one different phosphopeptide/protein. Three-times reasoning means that two phosphopeptides/proteins are linked through two different phosphopeptides/proteins, but the existing relationships in one-time reasoning and two-time reasoning were excluded. Finally, a database of interacting proteins (DIP) consisting of the reported PPIs was used to evaluate the relationships discovered by DCAA by matching the relationships discovered by DCAA and the relationships recorded in the DIP (Xenarios et al., 2002).

## Results and Discussion

### Different Clustering Methods Reflect Similar Patterns of Change in Data

**Figure 3:**
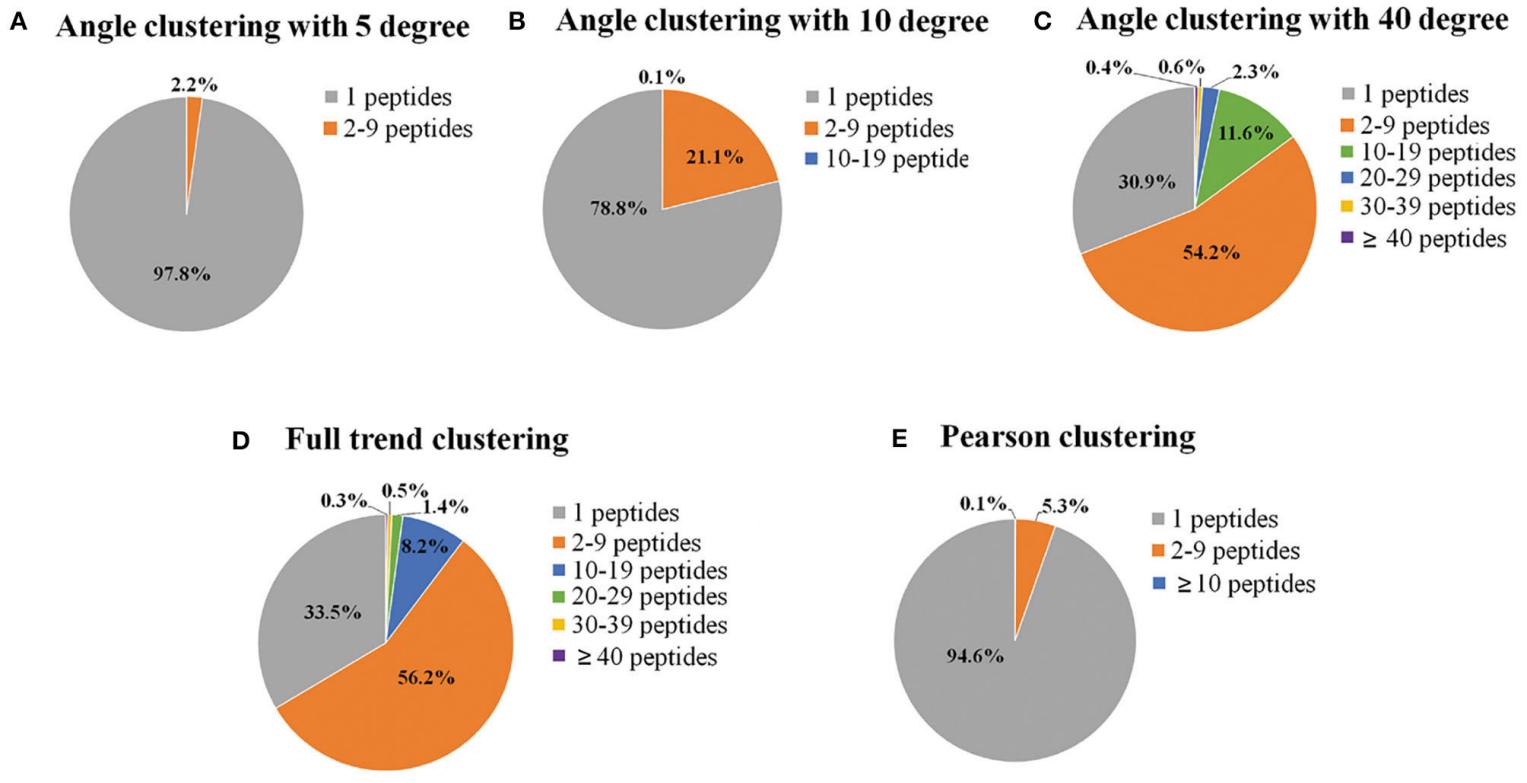
Pie charts of the percentages of clusters containing different numbers of phosphopeptides. The original time-series data of the relative expression levels of phosphorylated peptides were clustered, and then the percentage of the clusters containing different numbers of phosphopeptides was calculated by different clustering methods, including angle clustering at 5° **(A)**, 10° **(B)**, and 40° **(C)**, full trend clustering **(D)**, and Pearson clustering **(E)**. Full trend clustering: considering only the direction and not the extent of the changes. Pearson clustering: clustering based on Pearson's correlation coefficients.

In the present study, endothelial cells were seeded in T-175 flasks and treated with ox-LDL for different time periods to investigate the changes in protein phosphorylation. Four-hundred-microgram samples of proteins were taken from each time point to map phosphorylation sites using nano-LC-MS/MS. A total of 17,287 phosphorylation sites were identified on 15,037 phosphopeptides from 4,539 proteins. The dataset was used for mining the protein–protein interaction network. Each group contained 13 data points at different times (0.5, 1, 1.5, 2, 4, 6, 8, 12, 18, 24, 36, 48, and 72 h). To reduce the computational load of subsequent data processing, clustering was the first process of DCAA. Three different clustering methods, named full trend clustering, angle clustering, and Pearson clustering, were used to classify the original data set (Figure 3). The clustering algorithm in the paper can be regarded as the direct angle threshold method. For one group of data, 13 points can produce 12 line segments connected by two adjacent points. The 12 angles of the corresponding lines from the two groups of data were compared. If all of the angles are not larger than the threshold, they are classified into two classes. Physically, this indicates that the changes in every specific period between the two groups of data are similar. Angle clustering clustered the data groups with the same changing trend and similar changing speed. Therefore, angle clustering should divide the clusters of full trend clustering into smaller clusters. When the angle was set to 5°, 97.8% of clusters only contained one element class (only one peptide in the class), which implies failure of clustering (Figure 3A). When the angle was set to 10°, the percentage of clusters containing one peptide was significantly decreased (Figure 3B). When the angle was set to 40°, the percentages of clusters containing different amounts of peptides were similar to those of full trend clustering (Figures 3C,D). For Pearson clustering, different thresholds of the Pearson correlation coefficient (*R*) were tested on the original dataset. Most peptides with obvious relativities were put into one cluster when the threshold was set to 0.97. Therefore, we classify the data with |R| >0.97 as one cluster (Figure 3E). In sum, we obtained 3,494 clusters by full trend clustering, 12,300 clusters by angle clustering at 10°, and 13,686 clusters by Pearson clustering.

**Figure 4:**
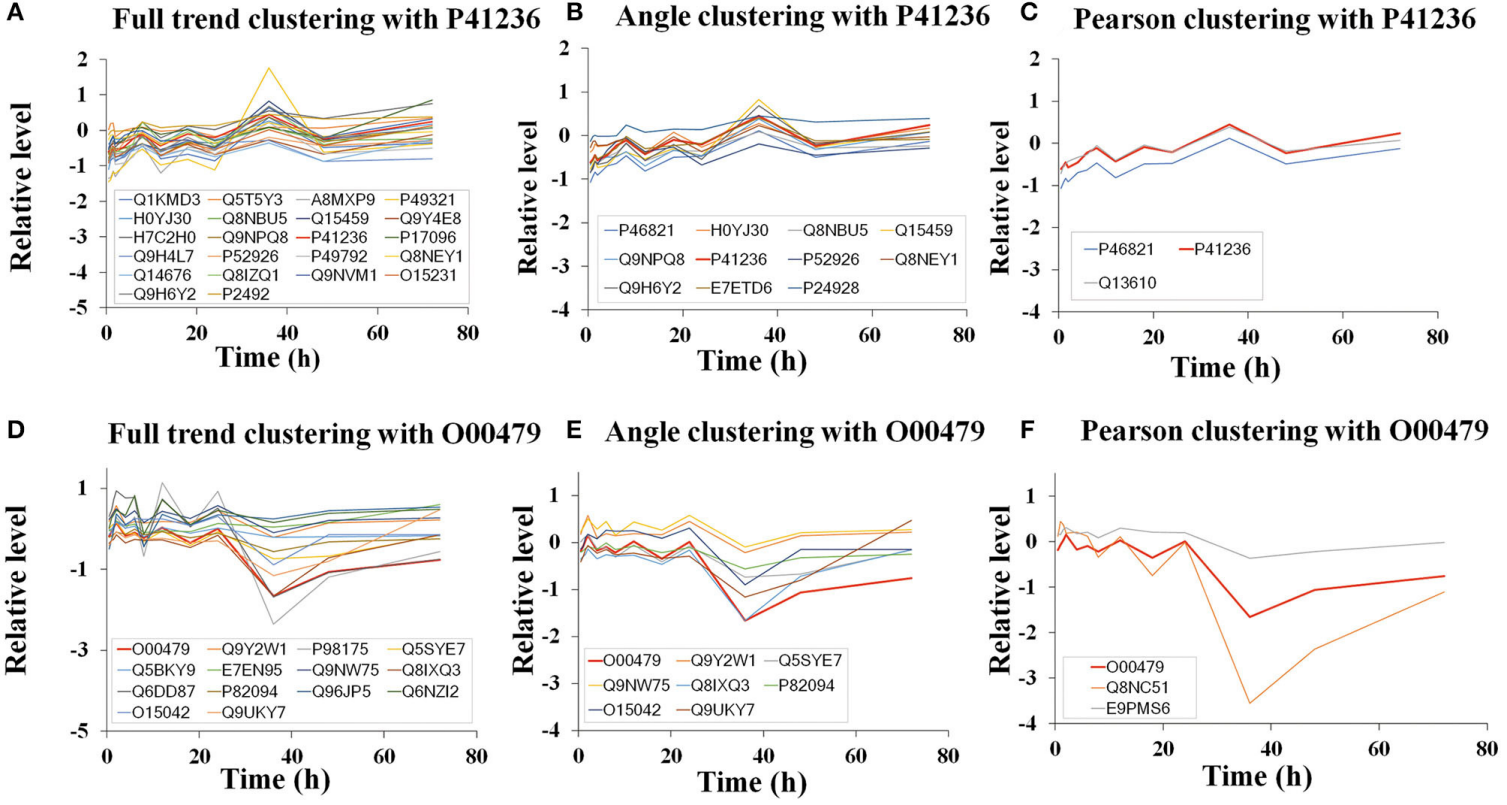
The representative results of clustering using different clustering methods. **(A–C)** The clusters obtained by different clustering methods, including full trend clustering **(A)**, angle clustering **(B)**, and Pearson clustering **(C)**. The trends of relative expression levels of the proteins in these clusters were consistent with P41236. **(D–F)** The clusters obtained by different clustering methods, including full trend clustering **(D)**, angle clustering **(E)**, and Pearson clustering **(F)**. The trends of the relative expression levels of the proteins in these clusters were consistent with O00479.

The indicators for evaluation of clustering algorithms included external and internal standards. External standards required knowing the previous distribution of samples. However, angle clustering at 10°, full trend clustering, and Pearson clustering are unsupervised clustering methods, so we evaluated them by internal standards. The internal standards were mainly based on the principle of inner-class distance and interclass distance. As Figure 4 shows, compared with angle clustering at 10° and Pearson clustering, full trend clustering had a higher degree of aggregation in the clustering of time-series data. The actual hit rates of interclass inference rules were obtained under different conditions for the aforementioned three clustering methods (Table 1). The average hit rate of full trend clustering was 68.7%, and full trend clustering was better than the other two clustering methods.

**Table 1:** The numbers of interclass inference rules using different minimum supporting degrees and confidence degrees.

**Full trend clustering**			**Angle clustering at 10**^****°****^			**Pearson clustering**		
**MSD**	**MCD**	**Rule numbers**	**MSD**	**MCD**	**Rule numbers**	**MSD**	**MCD**	**Rule numbers**
0.0054	0.9	0	0.026	0.9	0	0.020	0.9	2
0.0052	0.9	28	***0.024***	***0.9***	***528***	0.018	0.9	2
***0.0050***	***0.9***	***106***	0.022	0.9	566	0.016	0.9	78
0.0048	0.9	1,111	0.020	0.9	1,768	***0.014***	***0.9***	***210***
0.0046	0.9	2,252	0.018	0.9	2,094	0.012	0.9	427
0.0044	0.9	4,290	0.016	0.9	3,950	0.010	0.9	960
0.0042	0.9	11,136	0.014	0.9	5,168	0.008	0.9	3,263
0.0040	0.9	37,692	0.012	0.9	6,932	0.006	0.9	6,982
0.0038	0.9	74,941	0.010	0.9	11,223	0.004	0.9	17,641
0.0036	0.9	129,345	0.008	0.9	14,453	0.002	0.9	36,150

MSD, minimum support degree; MCD, minimum confidence degree.

*The bold and italic values indicates the most suitable MSD and MCD degree for different clustering*.

As examples, the clustering results for proteins P41236 and O00497 using the three clustering methods are shown in Figure 4. For protein P41236, 22, 11, and 3 peptides were placed into one cluster by full trend clustering, angle clustering at 10°, and Pearson clustering, respectively (Figures 4A–C). For protein O00497, 14, 8, and 3 peptides were placed into one cluster by full trend clustering, angle clustering at 10°, and Pearson clustering, respectively (Figures 4D–F).

### Evaluation of Different Clustering Methods of DCAA

**Figure 5:**
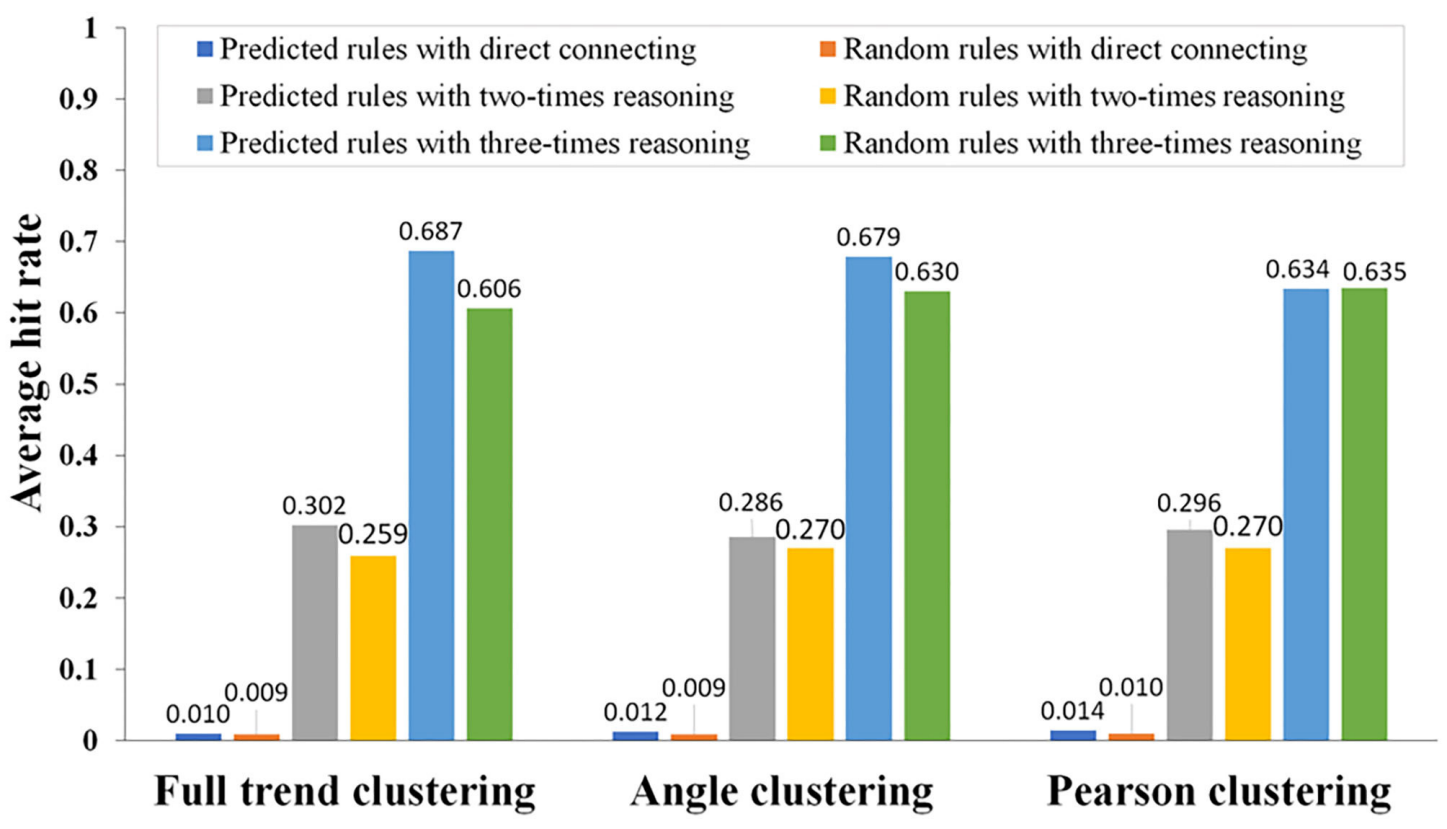
Comparison of the association rules predicted by DCAA and the random association rules using different reasoning modes. Using different clustering methods (direct connection, twofold reasoning, and threefold reasoning), the hit rates of association rules were calculated by comparing the predicted association rules with the association rules recorded in the DIP. Hit rates of DCAA and random matching were compared to judge whether there was a significant difference.

To evaluate different clustering methods used in DCAA, the DCAA hit rates for the association rules in the DIP were calculated by comparing the association rules based on each interclass inference rule obtained from DCAA with the records of association rules in the DIP. In addition, the same number of protein pairs as that in the association rules obtained from DCAA were randomly selected from the original dataset containing over 4,000 proteins, and the random association rules were created by randomly associating pairs of these proteins. Then, the random hit rates of association rules in DIP were calculated by comparing the random association rules with the records of association rules in DIP. The operation was repeated 100 times to obtain the average hit rate of random matching. Next, the DCAA hit rates of association rules in DIP were compared with the random hit rates of association rules. The calculation formulas of hit rates of DCAA and random matching were as follows:

(4) $$\begin{array}{l} R=\frac{m}{M} \end{array}$$

(5) $$\begin{array}{l} R^{\prime }=\frac{n}{M^{\prime }} \end{array}$$

where *m* is the number of association rules in the DIP predicted by DCAA and *n* is the number of randomly predicted association rules in the DIP. *M* and *M*′ are the number of predicted association rules and random association rules, respectively. The results showed that there were significant differences between the DCAA hit rates and the random hit rates (*p* < 0.01; Figure 5).

The three clustering methods used in this study were based on the shape similarity of time-series data, whereas popular approaches, including *k*-means and hierarchical clustering, measure the relevance between two clusters by distance. The methods based on distance ignore similar changing trends. However, the changing trends are important features of PPIs. Therefore, many peptides with similar changing trends but long distances are divided into different categories by clustering methods based on distance. Compared with these methods, full trend clustering, angle clustering, and Pearson clustering might be more suitable for PPI analysis.

### Types of Sliding Windows to Obtain Interclass Inference Rules

In the present study, a sliding time window was used for delayed comparison to reflect the lags between upstream and downstream events of PPIs. The size of the time window represents the affected time of delayed influence. Because the time of delayed influence in the test was not certain, many delayed comparisons with different time windows were executed. All possible delayed influences were attained by these tests. The type of delayed comparison was denoted as a combination of three integers connected by two dots. The first number indicated the length of data to be compared (the size of the sliding window). The start time and the length of the delayed time period were denoted by the second and third numbers, respectively. Some examples of delayed sliding window comparisons are shown in Table 2. The delayed comparison resulted in six datasets, which were merged into one dataset to construct the dataset for the Apriori algorithm. After processing with the Apriori algorithm, 3,494, 12,213, and 13,138 customer shopping records were obtained by full trend clustering, angle clustering, and Pearson clustering, respectively.

**Table 2:** Examples of sliding windows with different strategies.

**Type**	**Compared class**	**Time (hours)**												
		**0.5**	**1**	**1.5**	**2**	**4**	**6**	**8**	**12**	**18**	**24**	**36**	**48**	**72**
9.1.1	Class one	−0.11	−0.21	−0.33	−0.05	−0.01	−0.27	0.06	0.54	0.07	−0.05	−0.21	−0.37	−1.05
	Class two	−0.15	−0.11	−0.08	−0.12	−0.24	−0.25	−0.18	−0.21	−0.32	−0.54	−0.68	−0.28	−0.19
9.1.2	Class one	−0.11	−0.21	−0.33	−0.05	−0.01	−0.27	0.06	0.54	0.07	−0.05	−0.21	−0.37	−1.05
	Class two	−0.15	−0.11	−0.08	−0.12	−0.24	−0.25	−0.18	−0.21	−0.32	−0.54	−0.68	−0.28	−0.19
9.1.3	Class one	−0.11	−0.21	−0.33	−0.05	−0.01	−0.27	0.06	0.54	0.07	−0.05	−0.21	−0.37	−1.05
	Class two	−0.15	−0.11	−0.08	−0.12	−0.24	−0.25	−0.18	−0.21	−0.32	−0.54	−0.68	−0.28	−0.19
9.2.1	Class one	−0.11	−0.21	−0.33	−0.05	−0.01	−0.27	0.06	0.54	0.07	−0.05	−0.21	−0.37	−1.05
	Class two	−0.15	−0.11	−0.08	−0.12	−0.24	−0.25	−0.18	−0.21	−0.32	−0.54	−0.68	−0.28	−0.19
9.2.2	Class one	−0.11	−0.21	−0.33	−0.05	−0.01	−0.27	0.06	0.54	0.07	−0.05	−0.21	−0.37	−1.05
	Class two	−0.15	−0.11	−0.08	−0.12	−0.24	−0.25	−0.18	−0.21	−0.32	−0.54	−0.68	−0.28	−0.19
9.3.1	Class one	−0.11	−0.21	−0.33	−0.05	−0.01	−0.27	0.06	0.54	0.07	−0.05	−0.21	−0.37	−1.05
	Class two	−0.15	−0.11	−0.08	−0.12	−0.24	−0.25	−0.18	−0.21	−0.32	−0.54	−0.68	−0.28	−0.19

*By comparing the situation before and after sliding, a sliding window with different strategies could reflect the lags between upstream and downstream events of PPIs. The same color indicates the classes to be compared*.

Because too many rules could drastically increase the computational load, and too few rules may not support sufficient reasoning, the number of rules should be controlled at a proper level. In this paper, the number of rules was controlled to a few hundred. As the confidence degree mainly affected the probability of rule occurrence, we fixed the confidence degree at 0.9 and only adjusted the value of the support degree. Equally spaced values of support degree were tested, and the numbers of obtained rules are shown in Table 1. When using full trend clustering, 106 interclass inference rules were produced at a minimum support degree of 0.005. When using angle clustering at 10°, 528 interclass inference rules were obtained at a minimum support degree of 0.024. When using Pearson clustering, 210 interclass inference rules were obtained at a minimum support degree of 0.014 (Supplementary Table 1).

### The Matching Process of DCAA and Its Comparison With PPI Records in the DIP

**Figure 6:**
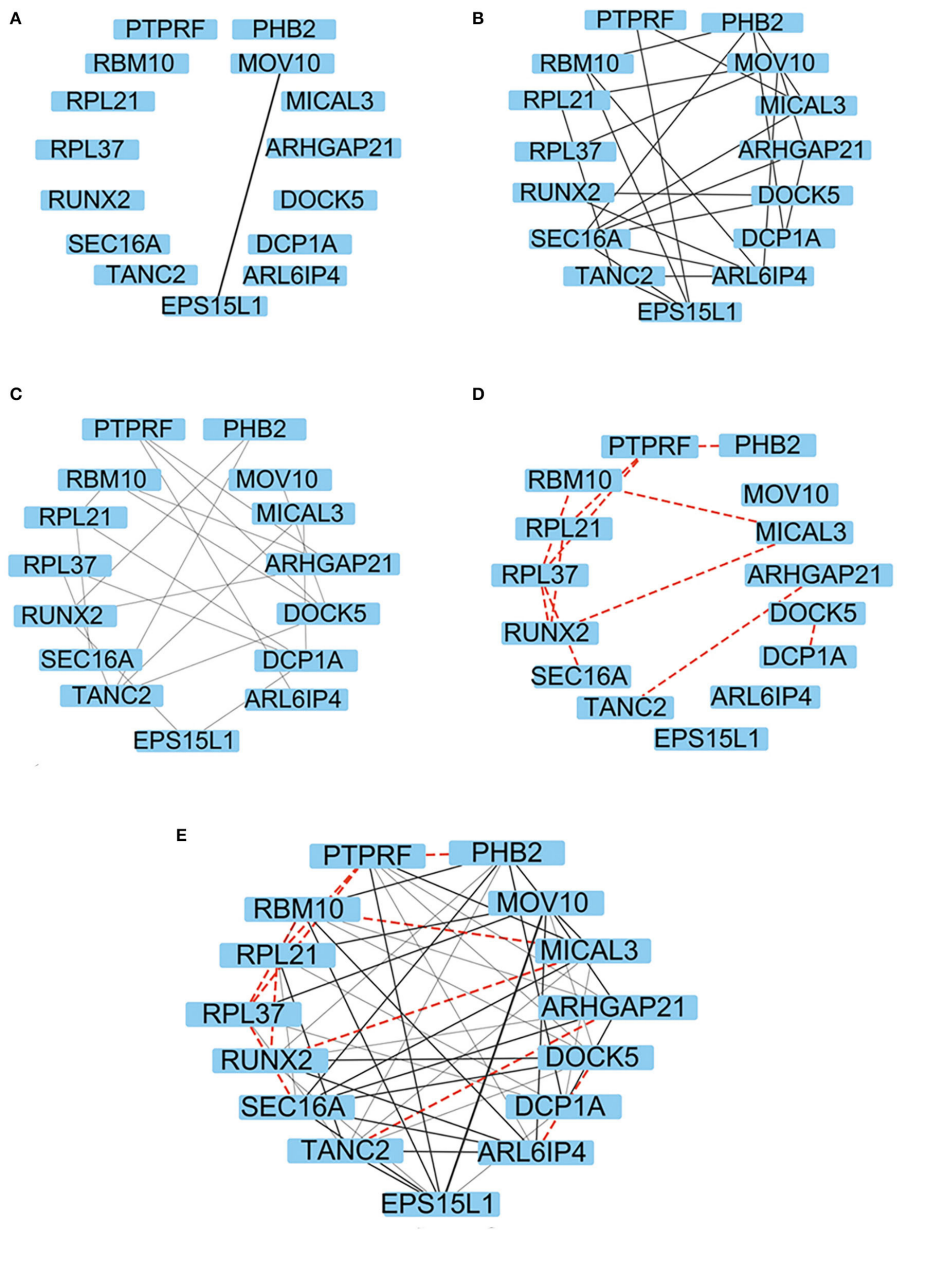
The representation of the association rules/PPIs revealed by DCAA. **(A–C)** The representation of the PPIs recorded in the DIP revealed by DCAA using different reasoning modes, namely, direct connection **(A)**, twofold reasoning **(B)**, and threefold reasoning **(C)**. **(D)** Representation of the PPIs that were predicted by DCAA but were not recorded in the DIP. The black solid lines represent the PPIs recorded in the DIP. The red dashed lines represent the PPIs predicted by DCAA. The thickness represents the distance of the relationship; the thicker the line is, the closer the relationship is **(E)** Representation of PPIs that were combined with the results of **(A–D)**.

The association rules identified by DCAA were compared with the reported association rules in the DIP. Figure 6 shows an example of the matching results. Figures 6A–C represents the inference results identified from the DIP under the conditions of direct connection, two-times reasoning, and three-times reasoning, respectively. Figure 6D shows the association rules that were predicted by DCAA but were not recorded in the DIP. These newly discovered PPIs should be helpful for designing biological experiments.

## Conclusions

**Figure 7:**
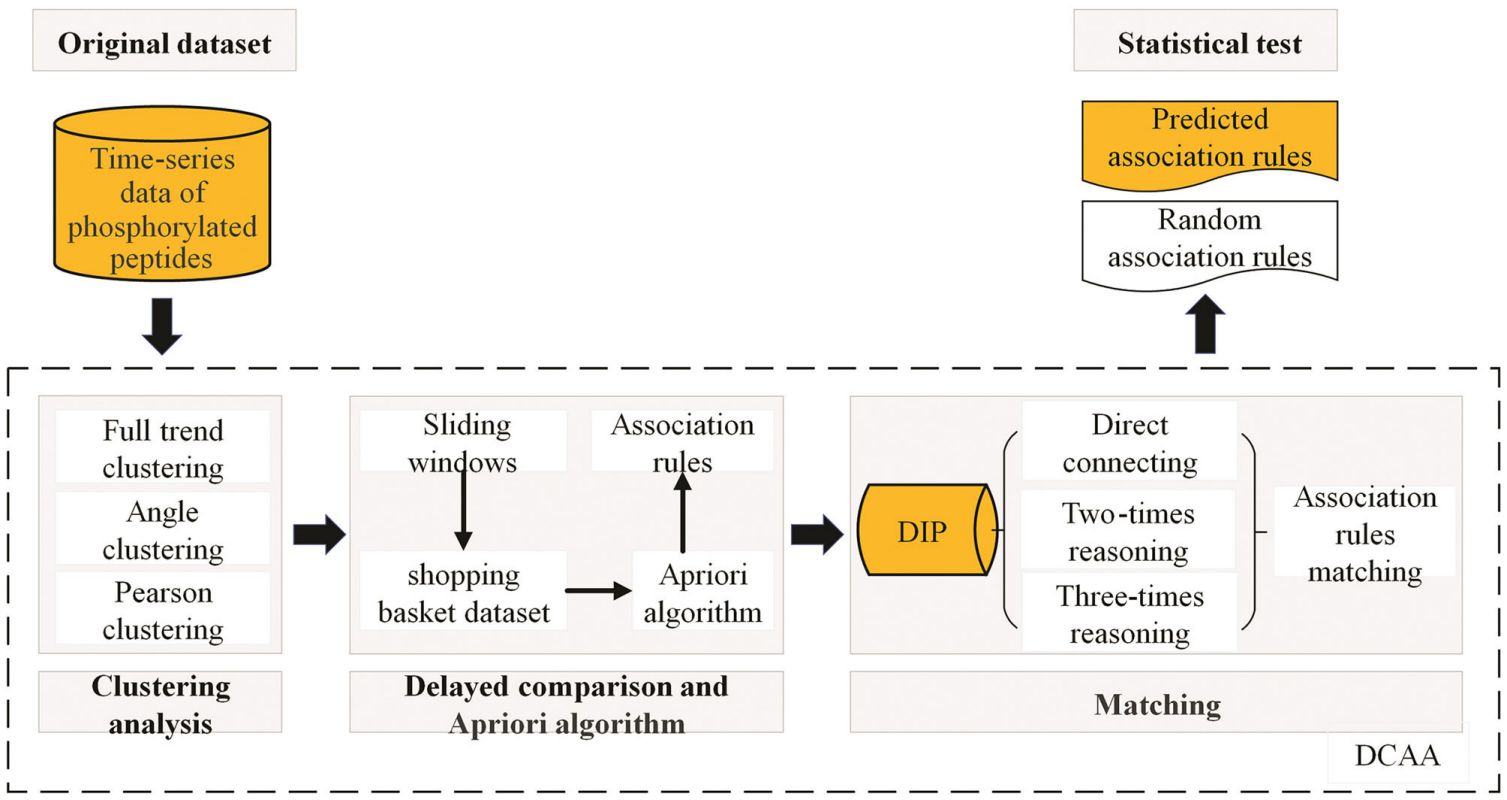
The procedure for the DCAA method. Clustering using different methods was the first step in DCAA analysis. After clustering, the delayed comparison was constructed by using sliding time windows to cover different situations involved in the hysteresis effect. Then, the Apriori algorithm was used to discover association rules for the potential PPIs. Next, the association rules predicted by DCAA and random linking were matched to the association records in the database of interacting proteins (DIP), and the hit rates were calculated. Finally, the hit rates of DCAA and random linking were compared to evaluate the prediction accuracy.

In the present study, a novel tool, DCAA, was developed to discover PPIs from time-series phosphoproteomic data. The basic idea of DCAA was to classify the peptides with similar changing trends into one class by clustering and then identify the association rules among different classes by delayed comparison and the Apriori algorithm. DCAA consists of three main steps, namely, clustering, delayed comparison, and the Apriori algorithm, as well as matching (Figure 7). In DCAA, the lags between upstream and downstream events of PPIs were considered. Therefore, DCAA can find novel association rules of proteins with relatively lower false-positive rates without previous knowledge and databases. DCAA should be useful to predict PPIs from time-series omics data, which is not limited to phosphoproteomic data.

## Data Availability Statement

The original contributions presented in the study are included in the article/Supplementary Materials, further inquiries can be directed to the corresponding author/s.

## Author Contributions

LD, HS, and HZ developed the algorithm, SX, SZ, and DL completed the experiment, PS, LC, and QZ designed the project, interpreted the data, and wrote the paper. All authors participated in writing the paper.

## Conflict of Interest

The authors declare that the research was conducted in the absence of any commercial or financial relationships that could be construed as a potential conflict of interest.
