# Supplementary material for: Delayed Comparison and Apriori Algorithm (DCAA): A Tool for Discovering Protein–Protein Interactions From Time-Series Phosphoproteomic Data
Source: Front Mol Biosci. 2020 Dec 10;7:606570. doi: 10.3389/fmolb.2020.606570 (PMC7758479; doi:10.3389/fmolb.2020.606570)
Supplement: Supplementary file 2 [file Table_2.DOCX]

**Appendix 1**

Algorithm: Pseudocode of Apriori algorithm

$$\begin{matrix} data:\text{ }A\text{ }dataset\text{ }of\text{ }itemsets \\ L_{\text{n}}:\text{ }Frequent n-itemsets \\ C_{\text{n}}:Candidate n-itemsets \\ x:An\text{ }itemset \\ minsupport:\text{ }Minimum\text{ }Support \\ i\leftarrow1; \\ Scan data to\text{ }get L_{i}; \\ while L_{i}\neq\emptyset\text{ } do \\ C_{i+1}\leftarrow Extend(L_{i}); \\ L_{i+1}\leftarrow\emptyset; \\ For x\in C_{i+1} do \\ If support(x)\geq\mathrm{minsupport} \mathrm{then} \\ L_{i+1}\leftarrow L_{i+1}\cap x; \\ \mathrm{end} \mathrm{if} \\ \mathrm{end} \mathrm{for} \\ \mathrm{i}\leftarrow\mathrm{i}+1; \\ \mathrm{end} \mathrm{while} \\ \\ Notes: \\ Extend(Li)\text{ }is\text{ }the\text{ }function\text{ '}Candidate\text{ }itemset\text{ }generation\text{ }procedure' \\ stated\text{ }in\text{ }\left(34\right). \\ Support(x)\text{ }returns\text{ }the\text{ }support\text{ }of\text{ }the\text{ }itemset x. \\ A\text{ }frequent n-itemset\text{ }is\text{ }the n-itemset\text{ }support\text{ }is\text{ }higher \\ than minsupport. \end{matrix}$$
